# Differential Responses of Community Biomass Stability to Short-Term Nitrogen and Phosphorus Inputs in Two Dominant Plant Communities of the Songnen Grassland, Northeast China

**DOI:** 10.3390/plants15152343

**Published:** 2026-07-30

**Authors:** Jian Liu, Yuqi Zhang, Yang Gao, Hongzhu Yu

**Affiliations:** Jilin Academy of Agricultural Sciences (Northeast Agricultural Research Center of China), Changchun 130033, China; liujan556677@163.com (J.L.); zhangyuqi2807@163.com (Y.Z.)

**Keywords:** grassland restoration, nitrogen and phosphorus input, biomass stability, enzyme activity

## Abstract

The Songnen region in northeastern China contains one of the world’s three largest area of soda saline–alkali soils. Its grasslands are experiencing severe salinization and nutrient impoverishment, which threaten regional ecological security. While nitrogen (N) and phosphorus (P) additions are widely applied to restore degraded grasslands, how the biomass stability of different dominant plant communities responds to such inputs remains unclear. Here, we compared two representative communities—one dominated by *Leymus chinensis* and the other by *Puccinellia distans*—under four treatments: control, N addition (15 g N m^−2^), P addition (15 g P_2_O_5_ m^−2^), and combined N + P addition. N and P additions increased species dominance in both communities, yet diversity and evenness declined in *L. chinensis* and increased in *P. distans*. Aboveground biomass peaked under N + P (increasing by 146.82% and 82.16%, respectively). Critically, biomass stability showed opposing trajectories: it rose by 27.69% in *L. chinensis* under N + P, but fell by 50.90% in *P. distans* under N alone. Mantel tests and random forest analyses linked biomass stability in *L. chinensis* to multiple soil enzymes, nutrients, and plant diversity, whereas *P. distans* biomass stability was primarily regulated by microbial nitrogen and phosphorus. Structural equation modeling further indicated that nutrient inputs in *L. chinensis* triggered a complex soil microbial–enzymatic cascade that positively coupled plant biomass and soil nutrients with biomass stability, while diversity exerted a negative effect. In *P. distans*, biomass stability was negatively driven by soil nutrients and biomass, with enzyme activity playing a mediating role. These findings reveal contrasting biomass stability-regulation mechanisms and provide a mechanistic basis for targeted restoration of saline–alkali grasslands.

## 1. Introduction

Stability is defined as the capacity of an ecosystem to maintain its core structure and key functions in the face of environmental variation and external disturbance [1]. This capacity is fundamental to sustaining productivity, preserving biodiversity, and securing the continued delivery of ecosystem services [2]. Within grassland ecosystems, plant community biomass stability represents the cornerstone of broader system stability [3]. Achieving durable grassland biomass stability is therefore both a central goal of grassland restoration and a focal point of contemporary remediation research [4]. Grassland biomass stability is a complex property, jointly shaped by numerous natural and anthropogenic drivers [5]. Human activities, including grazing, fencing, mowing, and nutrient addition, readily perturb the state of degraded grasslands [6,7,8]. Among these, nutrient addition exerts the most direct control over soil nutrient function [9], then modulates interspecific competition, reconfigures community structure, and ultimately influences patterns of species diversity and resource allocation [10]. Consequently, nutrient addition is a primary external determinant of shifts in plant community biomass stability.

The effects of nutrient addition on biomass stability, however, remain debated. Some work indicates that nitrogen and phosphorus inputs can rapidly elevate grassland productivity and bolster community biomass stability via overyielding, thereby reinforcing ecosystem steady states [11]. In contrast, other studies report that inorganic nutrient loading in natural grasslands constrains niche dimensions, disrupts competitive equilibria among species, and promotes the ascendancy of a few dominant taxa [12]. Such shifts reduce community diversity and structural heterogeneity at broader scales, ultimately undermining plant community biomass stability. It has further been noted that nutrient enrichment is a major driver of plant diversity loss in grasslands, posing latent risks to both ecosystem function and biomass stability [13]. These divergent findings underscore that the impacts of nutrient addition are far from straightforward; assessments based solely on aboveground proxies, such as vegetation diversity, provide an incomplete picture of the underlying mechanisms [14]. In addition, the contrasting responses observed in plant communities are ultimately rooted in modifications to the soil environment. For instance, shifts in climate and soil conditions can profoundly influence species composition and biomass stability in grasslands, as documented on the Mongolian Plateau [15]. During the restoration of grasslands, soil nutrients are known to regulate herbaceous community biomass stability through a succession of stage-specific dominant factors, playing a critical role in sustaining ecosystem productivity and stability [16]. Nutrient additions exert their initial effects on the soil matrix, altering physicochemical conditions and directly modifying nutrient availability [17]. These belowground changes restructure plant–soil feedbacks, which in turn steer the differentiation of aboveground community structure and function.

The extracellular hydrolytic enzymes they mediate jointly drive plant–soil feedback processes [18], thereby exerting profound effects on community biomass stability. Numerous studies have shown that nutrient addition significantly influences soil enzyme activities in grasslands, with effects strongly modulated by vegetation type, degradation status, and nutrient type [19,20,21]. For instance, nitrogen addition can markedly elevate the activities of N-acetyl-β-D-glucosaminidase and urease [22]. Enhanced phosphorus availability following P addition coordinately regulates key enzymes associated with nitrogen and phosphorus cycling [23]. Shifts in soil enzyme activity signal that the soil ecosystem is experiencing external perturbation, and pronounced differences in the activities of carbon-cycling enzymes (invertase, β-1,4-glucosidase, polyphenol oxidase, and peroxidase), nitrogen-cycling enzymes (arylamidase), and phosphorus-cycling enzymes (alkaline phosphatase) have been documented across grassland soils under contrasting vegetation types [24]. Similar patterns have been observed in studies involving leguminous forages [25]. Existing research, however, has largely centered on correlative analyses between vegetation characteristics (e.g., productivity, diversity) and soil physicochemical properties (e.g., pH, salinity, nutrient content) [1,12], while the pivotal role of soil enzyme activities—a critical functional link between microbial activity and nutrient cycling rates—remains frequently overlooked in saline–alkali grasslands.

The Songnen Grassland represents one of the world’s three primary distribution regions of soda saline–alkali soils [26] and has long confronted severe challenges including soil salinization, nutrient impoverishment, simplified vegetation community structure, and declining ecosystem service provision [27,28]. Under the selective pressures imposed by high environmental heterogeneity, a characteristic vegetation pattern has emerged in this region, dominated by *L. chinensis* and *P. distans* [29]. *L. chinensis* functions as the dominant species of the native climax community [30], whereas *P. distans* serves as the principal dominant species during pioneer or degraded successional stages. The two species occupy markedly distinct ecological niches along the salinity–alkalinity gradient, and the intrinsic mechanisms underpinning community biomass stability differ fundamentally between them. Their comparative study therefore provides an ideal ecological model for disentangling the differential regulatory mechanisms by which “salinity–alkalinity–nutrient” cross-stress shapes community biomass stability. Accordingly, this study focuses on *L. chinensis* and *P. distans* communities in the Songnen saline–alkali grassland, aiming to explore the effects of short-term nitrogen and phosphorus addition on the biomass stability of these two distinct dominant plant communities, as well as the underlying vegetation-soil regulatory mechanisms. The findings of this study can deepen the mechanistic understanding of biomass stability maintenance and recovery in degraded grassland ecosystems and provide practical guidance for targeted ecological restoration of saline–alkali grasslands.

## 2. Results

### 2.1. Plant Diversity and Biomass

**Figure 1:**
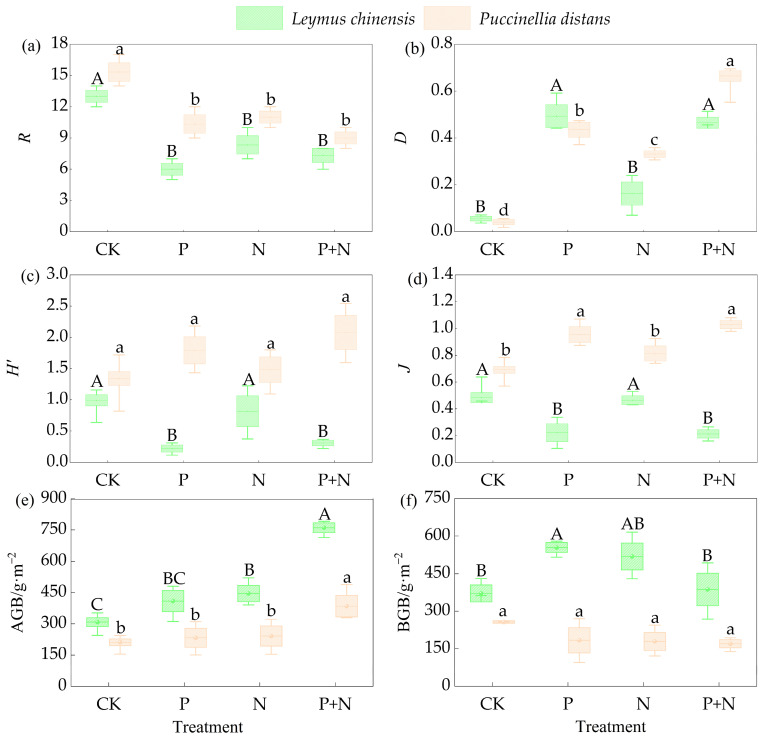
Plant diversity and biomass of different grassland communities under nitrogen and phosphorus additions. Values are presented as means ± standard error (SE). (**a**)species richness index (*R*); (**b**) Simpson diversity index (*D*); (**c**) Shannon–Wiener diversity index (*H′*); (**d**) Pielou’s evenness index (*J*); (**e**) aboveground biomass (AGB); (**f**) belowground biomass (BGB). Different uppercase letters indicate significant differences among treatments within the *L.chinensis* community, and different lowercase letters indicate significant differences among treatments within the *P. distans* community (Dunn’s test, *p* ≤ 0.05).

Nitrogen and phosphorus addition induced contrasting shifts in community structure between the two communities. In the *L. chinensis* community, species richness index (*R*), Shannon–Wiener diversity index (*H′*), and Pielou’s evenness index (*J*) all declined under nutrient addition, particularly under the P and N + P treatments (Figure 1). *R* reached its minimum under P, showing a 53.84% decrease relative to CK. *D* increased significantly under P and N + P, while *H′* and *J* declined significantly (*p* ≤ 0.05). In the *P. distans* community, *R* reached its minimum under N + P, showing a 40% decrease relative to CK. *D* rose significantly across all fertilization treatments (*p* ≤ 0.05), while *J* showed **a** significant increase under P and N + P treatments (*p* ≤ 0.05).

Aboveground biomass (AGB) increased significantly in both communities following nutrient addition, with the N + P treatment yielding the maximum values: 760 g m^−2^ in *L. chinensis* (a 146.82% increase) and 384.86 g m^−2^ in *P. distans* (an 82.16% increase) (Figure 1e). Belowground biomass (BGB) in *L. chinensis* peaked under P at 554.88 g m^−2^ (39.83% above CK), whereas BGB in *P. distans* showed no significant response to any treatment (*p* > 0.05).

### 2.2. Soil Physicochemical Properties and Enzyme Activities

In the *L. chinensis* community, soil bulk density (BD) showed no significant change under nutrient additions. In contrast, soil pH and salinity decreased significantly in all treated plots compared with the control (CK), with the lowest values (pH 8.66, salinity 0.052%) recorded under N + P (Table 1). Soil organic carbon (SOC) was significantly lower under P (4.62 g kg^−1^) and N + P (4.98 g kg^−1^), but remained unchanged under N alone. Total nitrogen (TN), total phosphorus (TP), microbial biomass carbon (MBC), microbial biomass nitrogen (MBN), and microbial biomass phosphorus (MBP) all reached their highest values under the P treatment alone (TN: 1.36 g kg^−1^; TP: 0.32 g kg^−1^; MBC: 174.73 mg kg^−1^; MBN: 13.27 mg kg^−1^; MBP: 5.21 mg kg^−1^), which were significantly higher than the control by 31%, 60%, 117%, 157%, and 38%, respectively (*p* ≤ 0.05).

In the *P. distans* community, soil bulk density (BD), pH, salinity, soil organic carbon (SOC), and total phosphorus (TP) showed no significant changes under nutrient addition (Table 2, *p* > 0.05). In contrast, total nitrogen (TN) increased significantly in all fertilization treatments (*p* ≤ 0.05), with the highest value under N alone (0.75 g kg^−1^, a 41.5% increase relative to CK). Microbial biomass nitrogen (MBN) and microbial biomass phosphorus (MBP) responded exclusively to N addition, reaching 6.61 mg kg^−1^ and 2.91 mg kg^−1^, respectively, which were 50.7% and 38.6% higher than the control (*p* ≤ 0.05). Microbial biomass carbon (MBC) remained unaffected across all treatments (*p* > 0.05).

Nutrient addition broadly elevated soil enzyme activities in both communities, with the notable exception of alkaline phosphatase (APK), which declined under all treatments (Table 3 and Table 4). In the *L. chinensis* community (Table 3), APK activity decreased significantly under P and N + P (lowest at 15.51 nmol g^−1^ h^−1^, –25.3% vs. CK), while the N-alone effect was not significant. β-glucosidase (βG) showed no significant changes across treatments. Cellobiohydrolase (CBH), N-acetyl-β-glucosaminidase (NAG), and leucine aminopeptidase (LAP) increased most under N alone: CBH by 43.1%, NAG by 34.4%, and LAP by 71.1% (*p* ≤ 0.05). β-xylosidase (βX) peaked under N + P (7.36 nmol g^−1^ h^−1^, +66.5% vs. CK, *p* ≤ 0.05). In the *P. distans* community (Table 4), APK suppression was stronger, with the lowest value under N + P (*p* ≤ 0.05). βG, NAG, and βX increased significantly under N and N + P (*p* ≤ 0.05), with βX showing the largest relative increase under N alone. CBH more than doubled only under N (*p* ≤ 0.05), while P and N + P had no effect. LAP increased significantly under P and N (peaking at 79.55 nmol g^−1^ h^−1^ under N, +58.1% vs. CK), but not under N + P.

### 2.3. The Biomass Stability of Different Dominant Communities

**Figure 2:**
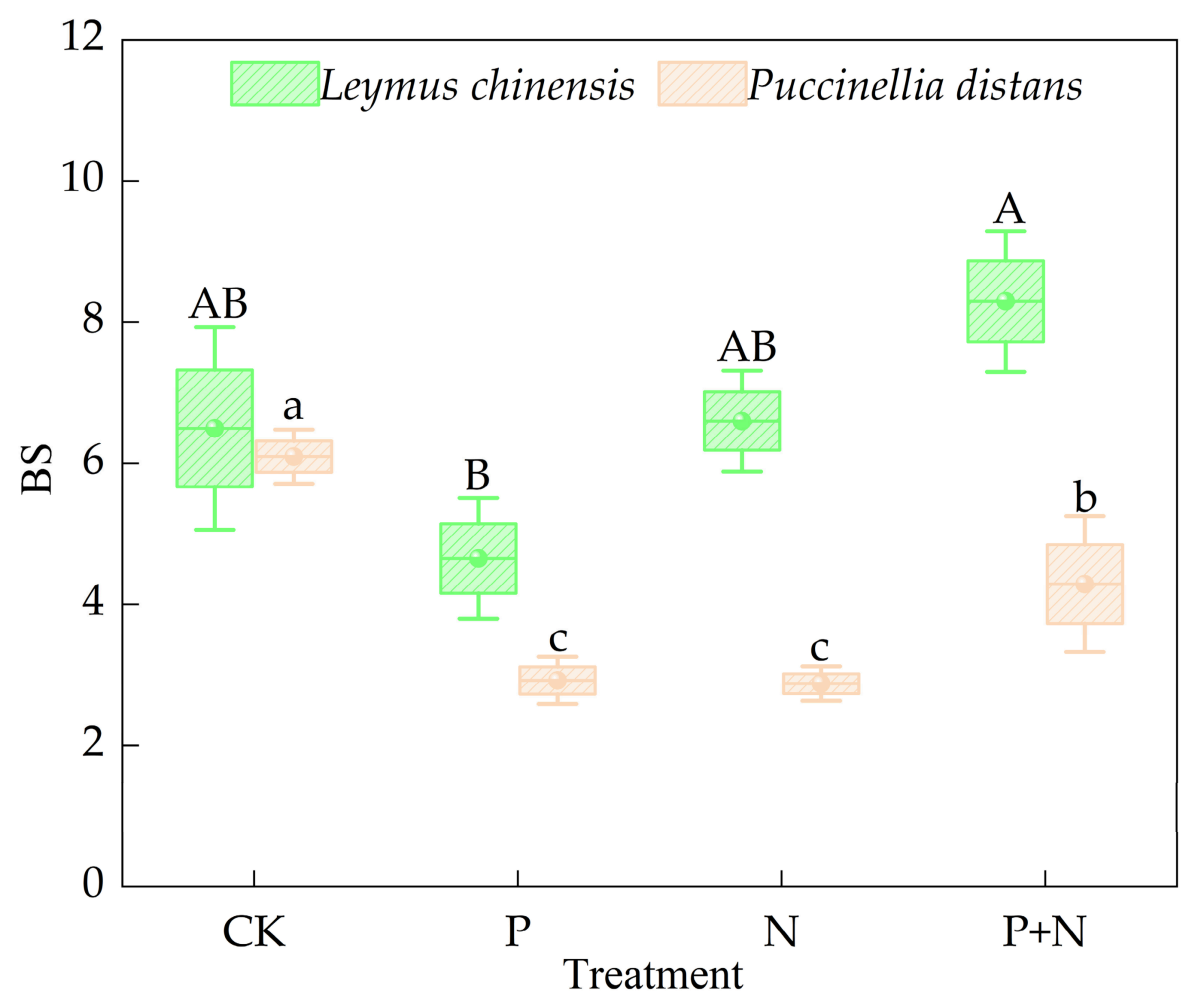
Biomass stability characteristics of different plant communities under nitrogen and phosphorus additions. Values are presented as means ± standard error (SE). BS, biomass stability. Different uppercase letters indicate significant differences among treatments within the *L. chinensis* community, and different lowercase letters indicate significant differences among treatments within the *P. distans* community (Dunn’s test, *p* ≤ 0.05).

The two communities displayed contrasting biomass stability responses to nutrient addition (Figure 2). In the *L. chinensis* community, biomass stability (BS) declined significantly under P addition alone (4.65, a 28.41% decrease relative to CK), yet increased under both N and N + P treatments, reaching its maximum (8.29) under combined N + P addition, showing a 27.69% improvement over CK. The *P. distans* community showed the opposite trend: biomass stability decreased under all nutrient treatments, with the sharpest decline occurring under N addition.

### 2.4. Effects of Community and Soil Characteristic Indicators on Grassland Biomass Stability

**Figure 3:**
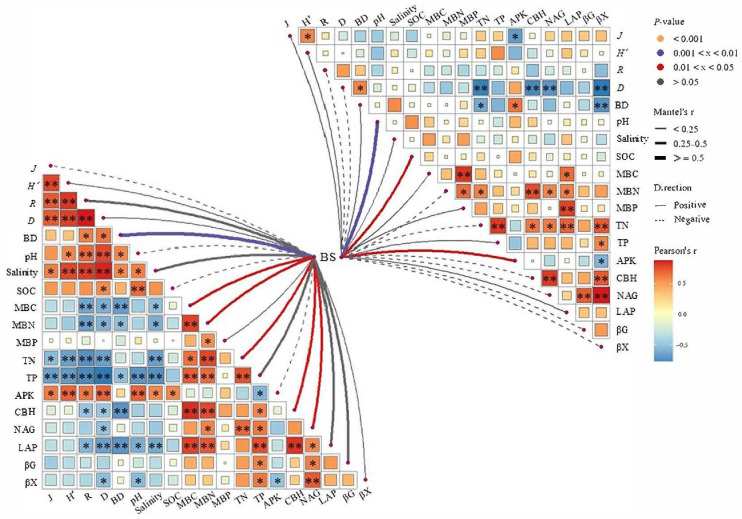
Correlations between grassland plant community biomass stability and various environmental factors based on Mantel tests. BS: biomass stability. *R*: species richness index; *D*: Simpson diversity index; *H′*: Shannon–Wiener diversity index; *J*: Pielou’s evenness index; BD: bulk density; pH: soil pH value; Salinity: soil salinity: electrical conductivity; SOC: soil organic carbon; TN: soil total nitrogen; TP: soil total phosphorus; MBC: microbial biomass carbon; MBN: microbial biomass nitrogen; MBP: microbial biomass phosphorus; APK: alkaline phosphatase; βG: β-1,4-glucosidase; CBH: cellobiohydrolase; NAG: β-1,4-N-acetylglucosamine glucosidase; βX: β-1,4-xylosidase; LAP: leucine aminopeptidase. The left panel corresponds to the *L. chinensis* community, and the right panel to the *P. distans* community. Significance levels: * *p* ≤ 0.05; ** *p* ≤ 0.01.

The VS in the *L. chinensis* community exhibited significant positive correlations with BD, MBC, MBN, TN, CBH and NAG (Figure 3, *p* ≤ 0.05). Among these, the correlations between BS and BD were highly significant (*p* ≤ 0.01). In the *P. distans* community, BS was significantly positively correlated with pH, SOC, and APK (*p* ≤ 0.05), with the correlation between BS and pH reaching an extremely significant level (*p* ≤ 0.01).

**Figure 4:**
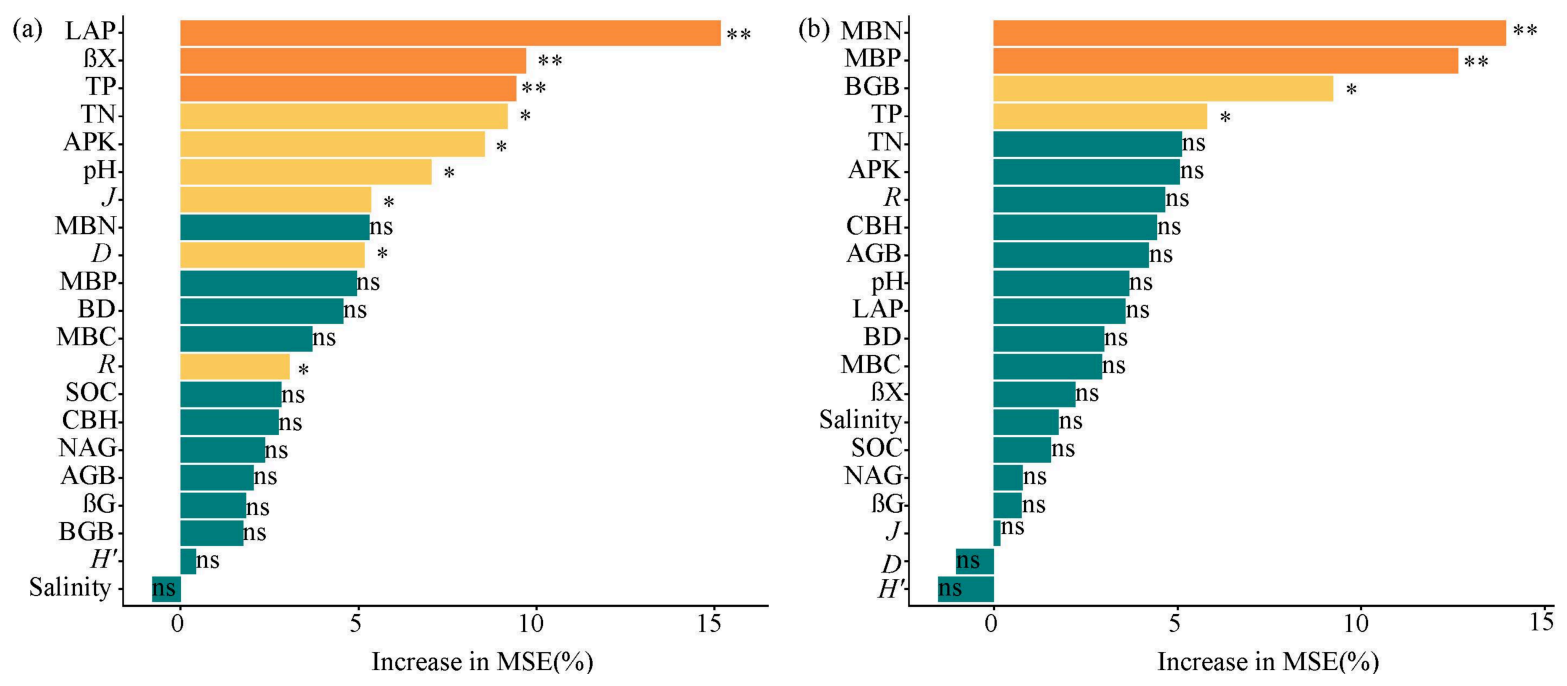
Relative importance of plant community characteristics and soil physicochemical properties to community biomass stability of *L. chinensis* (**a**) and *P. distans* (**b**). *R*: species richness index; *D*: Simpson diversity index; *H′*: Shannon–Wiener diversity index; *J*: Pielou’s evenness index; BD: bulk density; pH: soil pH value; Salinity: soil salinity: electrical conductivity; SOC: soil organic carbon; TN: soil total nitrogen; TP: soil total phosphorus; MBC: microbial biomass carbon; MBN: microbial biomass nitrogen; MBP: microbial biomass phosphorus; APK: alkaline phosphatase; βG: β-1,4-glucosidase; CBH: cellobiohydrolase; NAG: β-1,4-N-acetylglucosamine glucosidase; βX: β-1,4-xylosidase; LAP: leucine aminopeptidase. (**a**) *L. chinensis* community; (**b**) *P. distans* community. Significance levels: * *p* ≤ 0.05; ** *p* ≤ 0.01; ns *p* > 0.05.

The random forest model identified that LAP, *β*X, TP, TN, APK, pH, *J*, *D* and *R* contributed significantly to the BS of the *L. chinensis* community (*p* ≤ 0.05, Figure 4a). Among these variables, the increases in mean squared error (MSE) for LAP, *β*X, and TP were highly significant (*p* ≤ 0.01). In the *P. distans* community, MBN, MBP, BGB and TP made significant contributions to BS (*p* ≤ 0.05), with the MSE increases for MBN and MBP reaching a highly significant level (*p* ≤ 0.01; Figure 4b).

**Figure 5:**
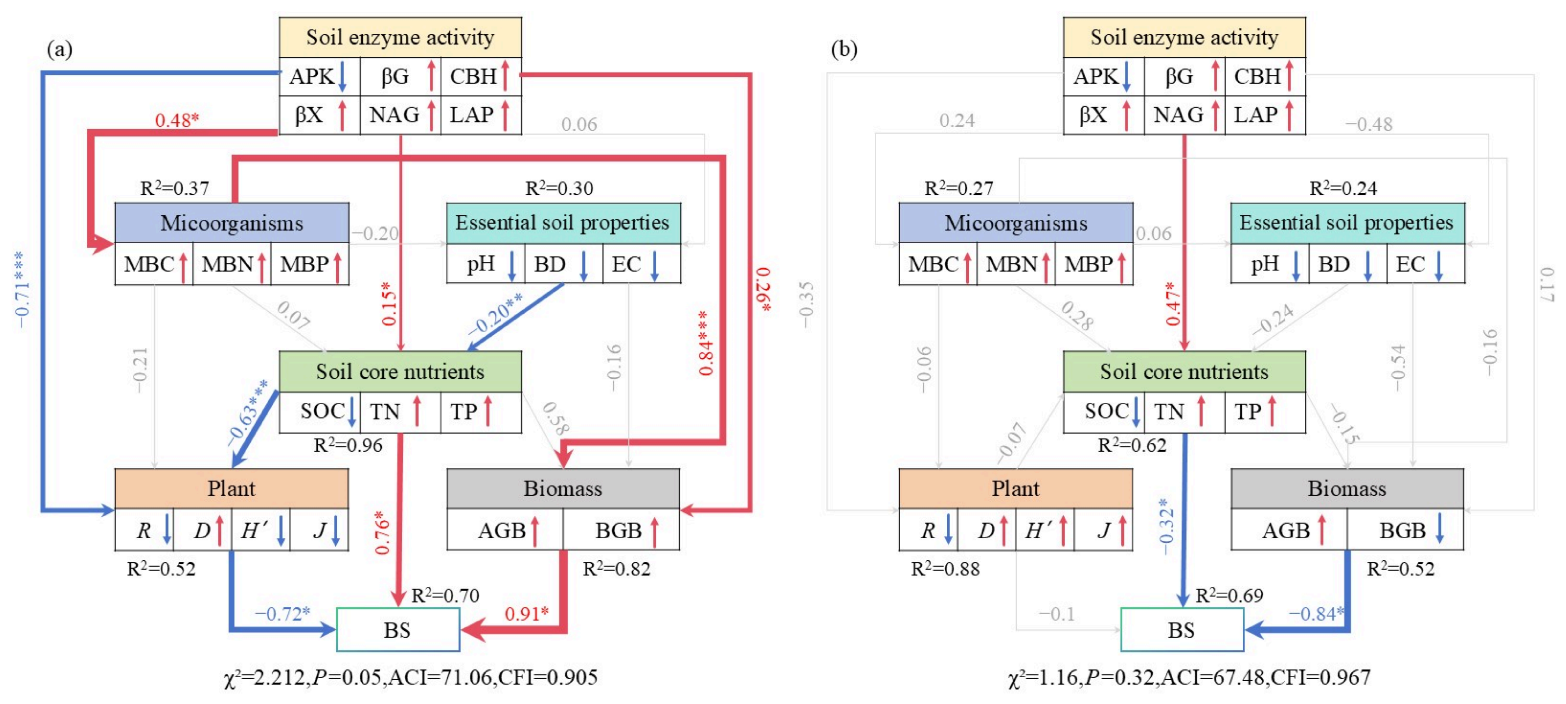
Structural equation modeling (SEM) revealing the direct and indirect effects of plant, soil, microbial properties, and specific enzyme activities on biomass stability in *L. chinensis* (**a**) and *P. distans* (**b**) communities. Single-headed arrows indicate the hypothesized direction of causation. Solid red and blue arrows denote positive and negative relationships, respectively, whereas gray arrows indicate non-significant relationships. Arrow width is proportional to the strength of the relationship. Multilayer rectangles represent the first principal component extracted from principal component analysis (PCA) conducted separately for plant properties, soil properties, microbial properties, and specific enzyme activities. The measured variables are organized into the following categories. Soil enzyme activities: alkaline phosphatase (APK), β-1,4-glucosidase (βG), cellobiohydrolase (CBH), β-1,4-N-acetylglucosamine glucosidase (NAG), β-1,4-xylosidase (βX), and leucine aminopeptidase (LAP). Essential soil properties: pH, bulk density (BD), and salinity. Microbial properties: microbial biomass carbon (MBC), microbial biomass nitrogen (MBN), and microbial biomass phosphorus (MBP). Core soil nutrients: soil organic carbon (SOC), soil total nitrogen (TN), and soil total phosphorus (TP). Plant community properties: species richness index (*R*), Simpson diversity index (*D*), Shannon–Wiener diversity index (*H′*), and Pielou’s evenness index (J). Plant properties: aboveground biomass (AGB) and belowground biomass (BGB). The red symbol (↑) and blue symbol (↓) indicate a positive or negative relationship between each measured variable and the first principal component of its respective PCA, respectively. Numbers adjacent to arrows are standardized path coefficients, reflecting the effect size of each relationship. The proportion of variance explained (R^2^) is shown alongside each response variable in the model. Goodness-of-fit statistics for the model are presented below each panel. Significance levels: * *p* ≤ 0.05; ** *p* ≤ 0.01; *** *p* ≤ 0.001. (For interpretation of the references to color in this figure legend, the reader is referred to the web version of this article).

Structural equation modeling (SEM) showed that soil enzyme activities (APK, βG, CBH, βX, NAG, LAP) influenced VS in the *L. chinensis* community through three distinct pathways (Figure 5a). First, soil enzyme activities exerted a strong negative effect on community diversity indices (*R*, *H*, *D*, *J*; *β* = −0.71, *p* ≤ 0.001), which in turn negatively affected VS (*β* = −0.72, *p* ≤ 0.05). Second, soil enzyme activities positively influenced soil core nutrients (SOC, TN, TP; *β* = 0.15, *p* ≤ 0.05), and these nutrients positively affected BS (*β* = 0.76, *p* ≤ 0.05); meanwhile, soil physicochemical properties (pH, BD, EC) negatively constrained soil core nutrients (*β* = −0.20, *p* ≤ 0.01). Third, soil enzyme activities directly and positively influenced biomass (AGB, BGB; *β* = 0.26, *p* ≤ 0.05), and also positively regulated soil microbial biomass (MBC, MBN, MBP; *β* = 0.48, *p* ≤ 0.05), which in turn positively affected biomass (*β* = 0.84, *p* ≤ 0.001); biomass ultimately showed a strong positive effect on BS (*β* = 0.91, *p* ≤ 0.05).

In contrast, the pathways influencing BS in the *P. distans* community were relatively simple. BS was significantly and directly negatively affected only by soil core nutrients and biomass (Figure 5b), and soil core nutrients showed a significant positive correlation with soil enzyme activity (*β* = 0.15, *p* ≤ 0.05).

## 3. Discussion

### 3.1. Contrasting Diversity–Biomass Trade-Offs in the Two Communities

Our results reveal that nitrogen and phosphorus additions altered species diversity in the two dominant communities of the Songnen saline–alkali grassland in opposite directions. In the *L. chinensis* community, species richness and diversity declined markedly under P and N + P treatments, accompanied by increased dominance; in contrast, the *P. distans* community showed no significant shifts in diversity, and N + P addition even resulted in marginally higher diversity than the control. The decline in *L. chinensis* diversity is consistent with the findings of Yang et al. [10] and can be explained by a nutrient-driven shift in competitive interactions: as soil nutrient availability increases, competition moves from belowground nutrient acquisition to aboveground light capture, accelerating competitive exclusion by the dominant species and simplifying community structure [31]. A global meta-analysis of long-term grassland fertilization experiments similarly demonstrated that nutrient addition, particularly combined N and P enrichment, reduces species richness primarily by elevating local extinction rates via competitive exclusion, rather than by impeding colonization [32]. This pattern supports the competitive exclusion hypothesis, whereby nutrient enrichment strengthens resource monopolization by dominant species and promotes increasingly uneven distributions of subordinate taxa [13]. Furthermore, multiple nutrient additions have been shown to homogenize the stoichiometric niches of coexisting species, intensifying competitive exclusion and further eroding plant diversity [33,34,35]. By contrast, the *P. distans* community displayed marked resistance to N and P inputs, a response likely rooted in the ecological strategies of pioneer species. Communities at early successional stages often consist of species with broader niche breadths and greater functional complementarity, which can buffer short-term resource fluctuations [36]. Long-term experiments in nutrient-poor pioneer grasslands also indicate that low to moderate nutrient inputs elicit only limited diversity responses, and significant diversity loss occurs primarily under high-dose N application [37]. The divergent diversity responses of the two communities thus fundamentally reflect differences in successional stage and competitive strategy along the salinity–alkalinity gradient, offering key insight into the mechanisms that regulate grassland biomass stability during saline–alkali grassland recovery.

The patterns of biomass allocation further underscore distinct resource-use strategies. Aboveground biomass in *L. chinensis* increased significantly under P and N + P treatments, with both communities achieving maximum aboveground biomass under N + P addition (a 146.82% increase over the control in *L. chinensis*). This indicates that both communities are subject to co-limitation by nitrogen and phosphorus, consistent with observations from alpine meadows on the Qinghai-Tibetan Plateau [38], and suggests that N and P addition rapidly alleviates pre-existing nutrient constraints, promoting biomass accumulation in clonal grass species and elevating community-level productivity. Above- and belowground biomass were higher in *L. chinensis* than in *P. distans* across all treatments, with the aboveground advantage particularly pronounced under P and N + P additions. This agrees with studies of successional recovery in degraded grasslands of northeastern China, which have shown that clonal *L. chinensis* populations possess superior rhizome storage capacity and nutrient-use efficiency, enabling more effective conversion of light and nutrient resources into biomass [39,40]. In the *P. distans* community, belowground biomass did not respond to nutrient addition. This contrasts with long-term observations in temperate grasslands, where nutrient addition commonly stimulates root growth [41]. A likely explanation is that *P. distans*, with its relatively short growing season and shallower rhizome system, maintains a root system already adapted to high-pH conditions; under improved nutrient availability, this pioneer species may preferentially allocate carbon to aboveground reproductive structures rather than investing in further root proliferation [42]. The contrasting biomass allocation patterns between the two communities thus reinforce the conclusion that *L*. chinensis and *P. distans* employ fundamentally different resource-use strategies in response to nutrient enrichment, shaped by their distinct successional positions and adaptive syndromes.

### 3.2. Differential Responses of Soil Physicochemical Properties, Nutrient Pools and Extracellular Enzymes to Short-Term N and P Addition in the Two Communities

Short-term nutrient addition significantly reduced soil pH and salinity with no detectable change in soil bulk density within mild-saline *L. chinensis* steppe. This observation is consistent with previous field experiments focusing on temperate grasslands under nitrogen enrichment [43]. The possible reasons are as follows: nutrient supply stimulated the growth of *L. chinensis*, which secreted certain amounts of low-molecular-weight organic acids into the rhizosphere. Together with rhizosphere microorganisms, these root exudates modulated ammonium nitrification and generated abundant hydrogen ions, ultimately decreasing bulk soil pH [44]. The increased plant biomass also strengthened root absorption of soluble salt ions, synchronously lowering soil salinity [45]. In contrast, no significant changes in soil pH or salinity were detected in the *P. distans* community. These muted physical responses are consistent with observations from peatland ecosystems [46], suggesting that *P. distans* naturally colonizes severely saline–alkali soils, whose restoration via phytodesalination requires long-term plant–soil interactions [47]. Its dense, shallow root mat covers topsoil to suppress surface water evaporation and block the upward capillary transport of soluble salts, thereby facilitating gradual salt leaching over multiple growing seasons. Nevertheless, the shallow-rooted trait limits its capacity to alter overall soil chemical properties within a short period; accordingly, nutrient addition exerted no significant influence on soil pH and salinity in the *P. distans* community [48]. Nutrient pools and microbial biomass, by contrast, revealed sharply different sensitivities. In the *L. chinensis* community, phosphorus proved to be the stronger driver. Phosphorus addition significantly increased soil total nitrogen, total phosphorus, microbial biomass carbon, microbial biomass nitrogen and microbial biomass phosphorus, whereas nitrogen addition only significantly elevated total phosphorus, microbial biomass carbon, and microbial biomass nitrogen, leaving total nitrogen and microbial biomass phosphorus unchanged. This uncoupled response points to phosphorus as the primary limiting element: when phosphorus supply is inadequate, soil microorganisms appear to channel acquired nitrogen toward their own growth and maintenance rather than driving synchronous turnover of the nitrogen and phosphorus pools [49]. The *P. distans* community displayed the opposite pattern—nitrogen was the dominant limitation. Only nitrogen addition produced pronounced effects on soil nutrients and microbial biomass; phosphorus addition triggered nothing beyond a marginal increase in total nitrogen, while total phosphorus and microbial biomass carbon, nitrogen, and phosphorus all held steady. Exogenous nitrogen thus relieves a core bottleneck in this community, stimulating microbial metabolic activity and promoting a positive nutrient cycling feedback [50,51]. A similar result was reported in alpine meadow ecosystems, where supplying the limiting nutrient shifted microbial functional gene composition and activated nutrient mineralization [31], reinforcing the broader principle that matching nutrient supplementation to local limitation substantially improves microbial activity and nutrient turnover efficiency.

Nitrogen and phosphorus additions did not uniformly elevate all hydrolytic enzymes; instead, carbon and nitrogen-acquiring enzymes (*β*G, *β*X, CBH, NAG, LAP) were significantly enhanced mainly under sole N or combined N + P treatments, whereas P alone rarely triggered significant increases. This pattern contrasts with previous reports that phosphorus addition broadly stimulates these enzymes in grasslands [52], and suggests that enzyme responses were driven primarily by nitrogen-mediated increases in plant-derived carbon inputs (litter, root exudates) rather than by SOC accumulation, which remained unchanged across treatments. APK activity displayed community-specific responses: in *P. distans*, APK changed significantly under each treatment; in *L. chinensis* it declined significantly under P and N + P, yet did not change significantly under N alone—an observation inconsistent with the generalized conclusion that N input universally suppresses APK [53]. A plausible explanation is that sole nitrogen addition elevates soil N:P ratios. Nitrogen enrichment stimulates plant and microbial growth and depletes available soil P, triggering microbial P starvation [54]. Microbes activate the *phoD* gene and enhance APK to mineralize organic P, thus maintaining stable APK abundance under N-only treatment. Field experiments on temperate *L. chinensis* steppe confirmed this trend: sole N addition increased soil N:P ratios without reducing the abundance of P-mineralizing functional genes, whereas P or combined N + P addition alleviated microbial P limitation and markedly lowered these gene abundances [55]. Nevertheless, the *P. distans* community exhibits a unique declining trend in APK under sole nitrogen supply, which contrasts sharply with the stable APK levels detected in *L. chinensis* communities. Such interspecific disparity can be mainly ascribed to the two dominant plant species and the unique rhizosphere microhabitats they mediate. Plants of different species establish divergent rhizosphere chemical environments, which fundamentally remodel phosphorus metabolic strategies of rhizosphere microbes [56]. For both *L. chinensis* and *P. distans* communities, phosphorus addition or combined nitrogen and phosphorus addition alleviates microbial phosphorus limitation and decreases microbial carbon allocation to APK synthesis for organic phosphorus mineralization. Coordinated downregulation of the phosphorus starvation-responsive gene *phoR* and upregulation of the low-affinity inorganic phosphate transporter gene *pit* may imply a potential metabolic shift in rhizosphere microbes in both species: under sufficient phosphorus supply, microbes may reduce APK-dependent organic phosphorus mobilization and prefer to directly take up, utilize and store inorganic phosphorus [57]. The two communities also differed in their carbon-cycling enzyme profiles. *β*G and CBH activities were consistently higher in *L. chinensis* than in *P. distans* across treatments, although P alone did not significantly elevate them, reflecting the inherently greater carbon turnover capacity of the climax community sustained by higher litter and rhizodeposits inputs [58,59]. This aligns with successional increases in βG activity reported by Lie et al. [60]. In contrast, NAG activity peaked under N addition in both communities, but the underlying drivers diverged. In the P-limited *L. chinensis* community, N addition still triggered a strong NAG response, indicating stimulated microbial N demand even when P is the primary limiting nutrient. In the N-limited *P. distans* community, N addition directly relieved N deficiency, maximizing NAG activity without requiring supplementary P. These contrasting NAG patterns confirm that microbial organic nitrogen utilization strategies are fundamentally shaped by the distinct nutrient limitation regimes determined by community degradation status [19].

### 3.3. The Driving Network of Major Factors Underlying Changes in Biomass Stability in L. chinensis Community

Under combined N + P addition, *biomass* stability of the *L. chinensis* community increased by 27.69%, a pattern consistent with observations from semi-arid Loess Plateau grasslands [4], where simultaneous alleviation of nitrogen and phosphorus limitation boosted productivity and possibly community evenness, thereby dampening the impact of resource fluctuations. In these saline–alkali systems co-limited by nitrogen and phosphorus, short-term nutrient input can rapidly relieve dual nutrient stress, and therefore maintain or even strengthen biomass stability via community structural optimization [61]. Diversity of *L. chinensis* communities exerted a significantly negative effect on *biomass* stability. This represents a short-term, specific outcome under nutrient enrichment conditions. Nutrient addition eliminates soil resource limitation, intensifies interspecific asymmetric competition, and weakens classical stabilizing mechanisms, including niche complementarity and functional redundancy. Meanwhile, the community fails to undergo adaptive structural succession over short experimental durations and cannot buffer productivity fluctuations through species functional compensation, ultimately reversing the traditional diversity–*biomass* stability relationship [62]. Notably, this finding does not negate the classical diversity–*biomass* stability hypothesis [63,64], but merely reflects the obvious environmental and scale dependence of this hypothesis. Diversity indices were constrained by soil enzyme activity and key nutrients. Nitrogen and phosphorus addition amplified the innate clonal propagation and competitive dominance of *L. chinensis*, enabling this species to rapidly displace subordinate plants and simplify community structure [65]. Collectively, both key nutrient enrichment and altered soil enzymatic activity reshape interspecific competition, thereby indirectly reducing community diversity indices. Although species richness declined markedly, the resulting highly dominated system displayed lower productivity variability—yielding a negative diversity–*biomass* stability link and reinforcing the view that, under enriched conditions, dominant species abundance, rather than richness per se, primarily governs *biomass* stability [66].

Key soil nutrients (SOC, TN, TP) and biomass (AGB, BGB) together had a strong direct positive effect on biomass stability (*β* = 0.91, *p* ≤ 0.01). Biomass stability is shaped by selection and complementarity effects, with selection prevailing when biomass derives largely from a single highly productive and competitive dominant [67]. Here, *L. chinensis* was the main contributor to community biomass; as short-term nutrient addition rapidly increased soil nutrients and productivity, it effectively buffered disturbances and dampened productivity fluctuations, thereby enhancing biomass stability. Biomass and soil nutrients were positively correlated with enzyme activities, and biomass also correlated positively with microbial biomass. These linkages mirror findings from degraded alpine meadows on the Qinghai-Tibetan Plateau [68], where nitrogen and phosphorus addition increased available nutrients, providing richer substrates for microorganisms. The positive coupling between microbial biomass and enzyme activity suggests that nutrient addition stimulated microbial activity and extracellular enzyme production, accelerating organic matter mineralization [69], improving nutrient cycling and soil quality, and indirectly promoting plant growth through positive plant–soil–microbe feedbacks [70].

### 3.4. The Simplified Core Pathway Underlies Biomass Stability Loss in the P. distans Community

In contrast, biomass stability of the *P. distans* community declined under nutrient addition, dropping most sharply (by 50.90%) with N alone. This likely reflects the comparatively weak response of *P. distans* itself to nitrogen enrichment. On the one hand, added N can amplify population fluctuations in this species [71]; on the other, N tends to favor more nitrogen-responsive competitors, intensifying interspecific competition and shifting the competitive hierarchy [72]. As a result, the regulatory role of the original dominant is weakened, and biomass stability declines [73]. In the *P. distans* community, the negative correlation between diversity and biomass stability was much weaker. Under less intense competition, short-term nutrient addition only modestly altered the competitive hierarchy, allowing subordinate species to retain broader niches. Changes in diversity thus covaried more tightly with productivity fluctuations, substantially weakening the expected negative correlation [14,58]. Moreover, biomass stability in this community was directly and negatively influenced only by soil core nutrients and biomass—a pattern opposite to the positive effects seen in *L. chinensis*. This suggests that in low-productivity systems like the *P. distans* community, the dominant species is not necessarily the most productive, so the contribution of selection effects to biomass stability is inconsistent. Instead, complementarity likely becomes the main mechanism reducing productivity variability. Under resource-poor conditions, the interplay between selection and complementarity often becomes nonlinear, producing a negative biomass–stability relationship [11].

Overall, the pathways regulating biomass stability in the *P. distans* community are considerably simpler than in *L. chinensis*, shaped largely by tight soil–vegetation coupling. The highly saline–alkali soil background restricts the availability of added nutrients and directly constrains microbial activity and enzyme function [74]. At the same time, restricted by short-term experimental limitations, the soil microbial community may undergo adaptive shifts, with metabolic and nutrient-transformation processes that respond only weakly to nitrogen and phosphorus inputs [75,76]. This also indicates that stable plant–soil feedbacks cannot be fully established in such high saline–alkali habitats within a short experimental period. Together, these factors dampen the positive feedbacks that nutrient addition might otherwise generate in the plant–soil system, leaving *P. distans* community biomass stability governed by a narrower set of regulatory pathways.

## 4. Materials and Methods

### 4.1. Study Area

The study was conducted at the Jiangjiadian Grassland, Da’an City, Jilin Province, located in the Songnen Plain of northeastern China. The central coordinates of the sampling plot are 45°16′ N, 123°34′ E, with an elevation of 136 m above sea level. The region has a temperate semi-humid continental monsoon climate. The mean annual temperature is 4.3 °C, the annual accumulated temperature (≥0 °C) is 2921.3 °C, and the annual sunshine duration is 3012.8 h. Mean annual precipitation is 460 mm, whereas annual evaporation reaches as high as 1800 mm. The area is characterized by windy and dry springs, concentrated rainfall in summer, clear and dry autumns, and long, cold winters.

The experimental plot has been fenced and excluded from grazing for more than ten years. The dominant plant species in the plot are *L. chinensis*, *P. distans*, *Phragmites australis*, and *Setaria viridis*. During the rainy season, the plot occasionally experiences temporary waterlogging, which typically lasts no longer than one week. The soil is classified as yellow sandy soil, with pH ranging from 7.9 to 9.5 and soil organic matter content of 1.22%. The baseline soil physicochemical properties of the two dominant community types selected for this study are presented in Table S1.

### 4.2. Experimental Design and Sampling

The experiment was conducted in May 2024 at the study site. Two grassland types—dominated by *L. chinensis* and *P. distans*, respectively—were selected for nitrogen (N) and phosphorus (P) addition treatments. The nitrogen fertilizer used in this experiment was urea (pure N content ≥ 46%), and the phosphorus fertilizer was superphosphate (available P_2_O_5_ content ≥ 12%). A completely randomized design was adopted with four treatments: control (CK, no N or P addition), P addition (P_2_O_5_, 15 g m^−2^, supplied by superphosphate), N addition (pure N, 15 g m^−2^, supplied by urea), and combined P + N addition (P_2_O_5_ 15 g m^−2^ + pure N 15 g m^−2^, supplied by superphosphate and urea respectively). Each treatment had three replicate plots, resulting in 12 plots per community type and 24 plots in total. Each plot measured 3 m × 5 m, with a buffer zone of at least 1 m between adjacent plots.

Sampling was conducted in late August 2024, corresponding to the peak growing season. Within each plot, three random sampling quadrats of 0.5 m × 0.5 m were established. For each plant species present in the quadrats, height (measured on three randomly selected individuals per species), number of individuals, and percent cover were recorded. Aboveground parts of all species were then clipped at ground level, separated by species, and collected into paper envelopes. In the laboratory, samples were oven-dried at 105 °C for 15 min, followed by drying at 80 °C to constant weight. The dry weight of each species was measured, and the sum of dry weights per quadrat was used to calculate aboveground biomass (AGB) per plot, expressed in g m^−2^. After harvesting aboveground biomass, belowground biomass (BGB) was determined from a soil core (10 cm diameter, 20 cm depth) collected from each plot. Roots were washed from the soil core, oven-dried to constant weight, and weighed to obtain BGB.

For soil physicochemical and microbial analyses, a soil auger (5 cm inner diameter) was used to collect soil samples from the 0–20 cm depth in each quadrat. Six cores per quadrat were pooled and thoroughly mixed, and visible stones and plant debris were removed. The mixed soil sample was then reduced using the quartering method into two subsamples. One subsample (fresh soil) was placed into sterile bags and stored at −40 °C for determination of MBC, MBN, MBP, and enzyme activities. The other subsample was air-dried at room temperature and passed through 10-mesh (2 mm) and 100-mesh (0.15 mm) sieves for routine physicochemical analyses.

### 4.3. Measurement of Soil Biotic and Abiotic Properties

SOC content was determined using the potassium dichromate-sulfuric acid external heating method [77]. TN content was measured by the Kjeldahl method using a Kjeltec 8400 analyzer (FOSS Analytical, Hillerød, Denmark) [78]. TP content was determined using H_2_SO_4_-HClO_4_digestion and analyzed by the molybdenum blue method using an ultraviolet spectrophotometer (T600/T700 Persee General Instrument Co., Ltd., Beijing, China) [79]. Soil pH was measured with a pH meter (PHS-25, Shanghai INESA Scientific Instrument Co., Ltd., Shanghai, China) at a soil-to-water ratio of 5:1 (*V*/*W*). Soil salt content was determined by the electrical conductivity method: soil was extracted at a soil-to-water ratio of 5:1, and the electrical conductivity of the extract was measured using a conductivity meter (DDS-307, Shanghai INESA Scientific Instrument Co., Ltd., Shanghai, China), which was then converted to soil salt content (g kg^−1^). Soil bulk density was measured using the cutting ring method: undisturbed soil cores were collected using a ring with a volume of 100 cm^3^, oven-dried at 105 °C to constant weight, and the mass of dry soil per unit volume was calculated.

MBC, MBN, and MBP were determined using the chloroform fumigation-extraction method [80]. After fumigation, soil samples were extracted with 0.5 mol L^−1^ K_2_SO_4_ for MBC and MBN analysis, and with 0.5 mol L^−1^ NaHCO_3_ for MBP analysis. SOC and TN contents in the extracts were determined using a total organic carbon (TOC) analyzer and the Kjeldahl method, respectively, whereas TP concentration was measured using the molybdenum-antimony ascorbic acid colorimetric method. All stoichiometric ratios of soil nutrients and available nutrients were calculated on a molar basis.

Soil enzyme activities were measured using a 96-well microplate fluorescence spectrophotometry. Six key enzymes involved in the transformation of soil carbon, nitrogen, and phosphorus were selected: βG, CBH,βX, NAG, LAP, and APK. The corresponding fluorogenic substrates were: 4-methylumbelliferyl-β-D-glucopyranoside (4-MUB-β-D-glucopyranoside) for βG; 4-methylumbelliferyl-β-D-cellobioside for CBH;4-methylumbelliferyl-β-D-xyloside for βX; 4-methylumbelliferyl-N-acetyl-β-D-glucosaminide for NAG; L-leucine-7-amino-4-methylcoumarin (Leu-AMC) for LAP; and 4-methylumbelliferyl-phosphate for ALP. Enzyme activities were expressed as nanomoles of fluorescent product produced per gram of dry soil per hour (nmol·g^−1^·h^−1^) [81].

### 4.4. Calculation of Community Characteristic Indices and Biomass Stability

*P_i_* = (RF + RD+ RC)/3 (1)

where $P_{i}$ is the importance value of species *i*, RF is the relative frequency; RD is the relative density; and RC is the relative coverage.

To compare species diversity among communities in terms of species richness, diversity, dominance and evenness, the Patrick richness index (R), Simpson diversity index (D), Shannon–Wiener diversity index ($H^{\prime }$), and Pielou evenness index (J) were calculated as follows [82]:

(2) R=S

(3) $$D=1-\sum_{i=1}^{S}P_{i}^{2}$$

(4) $$H^{\prime }=-\sum_{i=1}^{S}P_{i}lnP_{i}$$

(5) $$J=\frac{H^{\prime }}{\ln S}$$

where S is the number of species in a quadrat, $P_{i}$ is the importance value of species *i*.

Biomass stability (BS) was calculated as the ratio of the mean total biomass within each treatment ($\mu_{T}$) to its standard deviation within each treatment ($\sigma_{T}$) [1,2]:

(6) $$BS=\frac{\mu_{T}}{\sigma_{T}}$$

where $\mu_{T}$ represents the average total biomass under each fertilization treatment, ($\sigma_{T}$) is the standard deviation of total biomass for each treatment.

### 4.5. Data Analysis

To compare differences in plant properties, soil properties, enzyme activities, and community biomass stability among nutrient addition treatments within the same dominant community, we first assessed data normality using the Shapiro–Wilk test and homogeneity of variances using Levene’s test. After confirming that both assumptions were met, one-way analysis of variance (ANOVA) was performed, followed by Tukey’s honestly significant difference (HSD) test for post hoc pairwise comparisons. Statistical significance was set at *p* ≤ 0.05. The above analyses were conducted using SPSS version 20.0 (IBM Corp., Armonk, NY, USA). Boxplots plotted by Origin 2021(OriginLab Corporation, Northampton, MA, USA). Relationships between community biomass stability and individual plant and soil indicators were examined using Pearson correlation analysis and visualized with the corrplot package in R software (version 4.5.2). In addition, to evaluate overall multivariate associations, Mantel tests based on Euclidean distance matrices (9999 permutations) were conducted between the community biomass stability dissimilarity matrix and environmental dissimilarity matrices of plant and soil variables using the vegan package. Statistical significance for all analyses was set at *p* ≤ 0.05. A random forest model (RFM) was employed to evaluate the importance of various indicator traits for community biomass stability. The RFM was implemented using the randomForest package, and the significance of each predictor was assessed using the rfPermute package. All random forest analyses were performed using R software (version 4.5.2).

A structural equation model (SEM) was further employed to evaluate both the direct and indirect effects of plant community characteristics, soil physicochemical properties, soil nutrients, soil microbial properties, and soil enzyme activities on community biomass stability. To reduce multicollinearity among multiple variables within each group, principal component (PC) analysis was conducted [83]. The first principal component (PC1), which explained more than 50% of the total variance for each group, was considered credible as a composite variable representing the combined group properties in the SEM analysis [84]. An initial model was constructed using maximum likelihood estimation and subsequently refined by removing non-significant paths in a stepwise manner until an adequate model fit was achieved. Model fit was evaluated based on the chi-square (χ^2^) test, degrees of freedom (df), overall model *p*-value, root mean square error of approximation (RMSEA), and Akaike information criterion (AIC). SEM was implemented using AMOS 24.0 (Amos Development Corporation, Chicago, IL, USA).

## Figures and Tables

**Table 1:** Physical and chemical properties of soil in the *L. chinensis* community.

	CK	P	N	N + P
BD/g cm^−3^	1.49 ± 0.02 a	1.41 ± 0.01 a	1.40 ± 0.02 a	1.44 ± 0.03 a
pH	9.64 ± 0.51 a	8.69 ± 0.04 b	8.73 ± 0.17 b	8.66 ± 0.03 b
Salinity/%	0.0625 ± 0.002 a	0.054 ± 0.003 b	0.055 ± 0.004 b	0.052 ± 0.002 b
SOC/g kg^−1^	6.24 ± 0.16 a	4.62 ± 0.21 b	5.42 ± 0.30 ab	4.98 ± 0.46 b
TN/g kg^−1^	1.04 ± 0.13 c	1.36 ± 0.08 a	1.14 ± 0.06 bc	1.24 ± 0.10 ab
TP/g kg^−1^	0.20 ± 0.01 c	0.32 ± 0.01 a	0.29 ± 0.01 b	0.32 ± 0.03 a
MBC/mg kg^−1^	80.31 ± 5.13 b	174.73 ± 6.74 a	158.54 ± 7.32 a	140.69 ± 8.61 a
MBN/mg kg^−1^	5.17 ± 1.51 c	13.27 ± 0.84 a	9.86 ± 0.31 ab	8.18 ± 1.23 bc
MBP/ mg kg^−1^	3.77 ± 0.52 b	5.21 ± 0.24 a	3.83 ± 1.23 b	2.57 ± 0.37 b

Note: Different letters in the same row indicate significant differences among different treatments (*p* ≤ 0.05) using Dunn’s tests. BD, bulk density; SOC, soil organic carbon; TN, soil total nitrogen; TP, soil total phosphorus; MBC, microbial biomass carbon; MBN, microbial biomass nitrogen; MBP, microbial biomass phosphorus.

**Table 2:** Physical and chemical properties of soil in the *P. distans* community.

	CK	P	N	N + P
BD/g·cm^−3^	1.54 ± 0.03 a	1.50 ± 0.011 a	1.47 ± 0.02 a	1.46 ± 0.02 a
pH	9.93 ± 0.09 a	9.78 ± 0.29 a	9.90 ± 0.10 a	9.61 ± 0.55 a
Salinity/%	0.154 ± 0.016 a	0.159 ± 0.025 a	0.142 ± 0.020 a	0.130 ± 0.01 a
SOC/g kg^−1^	6.33 ± 0.54 a	5.55 ± 0.31 a	6.18 ± 0.31 a	5.56 ± 0.35 a
TN/g kg^−1^	0.53 ± 0.02 c	0.63 ± 0.04 b	0.75 ± 0.08 a	0.66 ± 0.04 ab
TP/g kg^−1^	0.21 ± 0.01 a	0.23 ± 0.01 a	0.26 ± 0.03 a	0.24 ± 0.02 a
MBC/mg kg^−1^	79.33 ± 7.96 a	106.50 ± 13.28 a	97.35 ± 7.54 a	76.46 ± 5.36 a
MBN/mg kg^−1^	4.42 ± 0.31 b	4.52 ± 0.19 b	6.61 ± 0.45 a	4.32 ± 0.42 b
MBP/mg kg^−1^	2.10 ± 0.19 b	2.64 ± 0.11 ab	2.91 ± 0.29 a	1.95 ± 0.21 b

Note: Different letters indicate significant differences among different treatments (*p* ≤ 0.05) using Dunn’s tests. BD, bulk density; SOC, soil organic carbon; TN, soil total nitrogen; TP, soil total phosphorus; MBC, microbial biomass carbon; MBN, microbial biomass nitrogen; MBP, microbial biomass phosphorus.

**Table 3:** Soil enzyme activity in the *Leymus chinensis* community.

	CK	P	N	N + P
APK/nmol g^−1^ h^−1^	20.77 ± 0.95 a	16.87 ± 72 b	17.91 ± 1.53 ab	15.51 ± 0.67 b
βG/nmol g^−1^ h^−1^	12.65 ± 0.47 a	13.66 ± 0.46 a	14.42 ± 0.22 a	14.94 ± 2.02 a
CBH/nmol g^−1^ h^−1^	3.83 ± 0.42 c	5.30 ± 0.30 b	5.48 ± 0.68 a	4.56 ± 0.19 bc
NAG/nmol g^−1^ h^−1^	2.09 ± 0.06 b	2.76 ± 0.15 a	2.81 ± 0.13 a	2.80 ± 0.14 a
βX/nmol g^−1^ h^−1^	4.42 ± 0.68 b	5.96 ± 0.50 ab	6.36 ± 0.52 a	7.36 ± 0.47 a
LAP/nmol g^−1^ h^−1^	71.72 ± 8.36 b	116.67 ± 2.41 a	122.7 ± 13.98 a	109.15 ± 12.69 a

Note: Different letters indicate significant differences among different treatments (*p* ≤ 0.05) using Dunn’s tests. APK, alkaline phosphatase; βG, β-1, 4-Glucosidase; CHB, cellobiohydrolase; NAG, β-1,4-N-acetylglucosamine glucosidase; βX, β-1, 4 Xylosidases; LAP, leucine aminopeptidase.

**Table 4:** Soil enzyme activity in the *P. distans* community.

	CK	P	N	N + P
APK/nmol g^−1^ h^−1^	16.97 ± 0.45 a	8.20 ± 0.93 bc	11.44 ± 1.43 b	6.01 ± 1.03 c
βG/nmol g^−1^ h^−1^	8.80 ± 0.21 b	8.62 ± 0.58 b	12.86 ± 0.85 a	13.96 ± 0.79 a
CBH/nmol g^−1^ h^−1^	2.59 ± 0.10 b	3.14 ± 0.40 b	5.99 ± 0.53 a	2.88 ± 0.57 b
NAG/nmol g^−1^ h^−1^	1.54 ± 0.13 c	1.93 ± 0.33 bc	3.08 ± 0.24 a	2.54 ± 0.15 ab
βX/nmol g^−1^ h^−1^	2.24 ± 0.22 c	5.88 ± 0.73 b	8.58 ± 0.83 a	6.37 ± 0.49 b
LAP/nmol g^−1^ h^−1^	50.33 ± 2.48 b	72.91 ± 6.11 a	79.55 ± 13.66 a	63.53 ± 6.52 ab

Note: Different letters indicate significant differences among different treatments (*p* ≤ 0.05) using Dunn’s tests. APK, alkaline phosphatase; βG, β-1, 4-Glucosidase; CHB, cellobiohydrolase; NAG, β-1,4-N-acetylglucosamine glucosidase; βX, β-1, 4 Xylosidases; LAP, leucine aminopeptidase.

## Data Availability

The original contributions presented in the study are included in the article. Further inquiries can be directed to the corresponding authors.

## Supplementary Materials

The following supporting information can be downloaded at: https://www.mdpi.com/article/10.3390/plants15152343/s1, Table S1: Pre-treatment Soil Physicochemical Characteristics of *L.chinensis* and *P. distans* Communities [85].
