# Supplementary material for: Differential Responses of Community Biomass Stability to Short-Term Nitrogen and Phosphorus Inputs in Two Dominant Plant Communities of the Songnen Grassland, Northeast China
Source: Plants (Basel). 2026 Jul 30;15(15):2343. doi: 10.3390/plants15152343 (PMC13468104; doi:10.3390/plants15152343)
Supplement: Supplementary file 1 [file plants-15-02343-s001.zip › plants-4408503-supplementary.pdf]

Differential Responses of Community Biomass Stability to Short-Term Nitrogen and Phosphorus Inputs in Two Dominant Plant Communities of the Songnen Grassland, Northeast China

Jian Liu, Yuqi Zhang, Yang Gao * and Hongzhu Yu *

Jilin Academy of Agricultural Sciences (Northeast Agricultural Research Center of China), Changchun 130033, China; gaoyang2302@126.com
* Correspondence: gaoyang2302@126.com; y22080@163.com

Rationale for Nutrient Addition Rates in This Experiment

the high N and P addition rates (15 g N m^{-2} and 15 g P_2O_5 m^{-2}) were justified by our prior field trials on Songnen degraded grasslands at Jiangjiadian (Liu et al., 2021). This reference reports our team's earlier published experiment conducted at the identical Jiangjiadian site, where their high-dose fertilization treatment (225 kg urea ha^{-1} + 135 kg diammonium phosphate ha^{-1}) delivers comparable nutrient input magnitudes to the current study. Our pre-experiment found moderate to high N and P inputs alleviated nutrient limitation, raised soil available nutrients and restored *Leymus chinensis* productivity. We applied this high dosage to identify the saturation threshold of community and soil responses to nutrients and clarify grassland functions under intense nutrient loading. This treatment matches the optimal fertilization regime from our long-term regional restoration tests and mimics cumulative nutrients from repeated fertilization and atmospheric deposition. Thus, the high nutrient levels were selected rationally according to local grassland restoration needs .

Table S1 Pre-treatment Soil Physicochemical Characteristics of *L.chinensis* and *P. distans* Communities

	BD /g·cm^{-3}	pH	Salinity/%	SOC/g kg^{-1}	TN/g kg^{-1}	TP/g kg^{-1}
Leymus chinensis community	1.5507	9.8500	0.0690	6.4074	0.9750	0.1927
Puccinellia distans community	1.6333	10.3300	0.0148	6.0157	0.5303	0.2179

Note：BD, bulk density; SOC, soil organic carbon; TN, soil total nitrogen; TP, soil total phosphorus

References

[1] Liu J, Gao Y, Wang Z F, et al. Effects of root cutting and fertilization on Leymus chinensis yield and soil fertility of degraded grassland in Jiangjiadian[J]. Heilongjiang Animal Science and Veterinary Medicine, 2021, (17): 96-103.
